# Effects of improved creative play interventions on social communication, behavioral, and cognitive function in children with autism spectrum disorder: a randomized controlled trial

**DOI:** 10.3389/fpubh.2026.1768848

**Published:** 2026-04-10

**Authors:** Mengchang Zhang, Mengzi Zhao, Yanfang Zhang, Fang Gao, Qing Zhao, Yanping Wang, Puyu Tian, Shaohua Zhang

**Affiliations:** Department of Pediatrics, The First People’s Hospital of Nanyang City, Nanyang, Henan, China

**Keywords:** ASD, cognitive functioning, improved creative play intervention, randomized controlled trial, social communication

## Abstract

**Background:**

Children with autism spectrum disorder (ASD) often exhibit deficits in social communication, repetitive behaviors, and cognitive delays. Play-based interventions have shown effectiveness in the treatment of ASD. This study aims to investigate the impact of improved creative play interventions combined with routine rehabilitation on these domains in autistic children.

**Methods:**

This single-blind randomized controlled trial enrolled 72 children aged 3–8 years with ASD. Participants were randomized to the control group (*n* = 36; routine rehabilitation) or intervention group (*n* = 36; routine rehabilitation + improved creative play intervention). Assessments were conducted at baseline and post-intervention (week 12) using the Aberrant Behavior Checklist (ABC), Autism Treatment Evaluation Checklist (ATEC), Childhood Autism Rating Scale-2 (CARS-2), Social Responsiveness Scale-2 (SRS-2), and the Chinese version of Psychoeducational Profile-3 (C-PEP-3) to measure changes in social skills, behavior, and cognitive functioning.

**Results:**

The intervention group showed greater improvements than the control group in the SRS-2 T-score (*p* < 0.001). No significant group × time interactions (*p* > 0.05) were found on aberrant behavior (ABC) or overall autism severity (CARS-2). Significant advantages in the intervention group were also observed across all four ATEC subscales and the total score (*p* < 0.001) compared with the control group. The improved creative play intervention group achieved significantly greater gains in fine motor, hand-eye coordination, cognitive performance, and verbal cognition domains of C-PEP-3, with particularly large differences in cognitive performance (*p* < 0.001).

**Conclusion:**

The improved creative play intervention combined with routine rehabilitation significantly enhances social, behavioral, and cognitive outcomes in ASD children.

## Introduction

Autism spectrum disorder (ASD) is a neurodevelopmental condition affecting approximately 1% of children worldwide (1). Children with ASD typically exhibit deficits in social communication and interaction, and restricted, repetitive behaviors and interests, often accompanied by developmental or cognitive delays, emotional dysregulation, and psychological challenges (2, 3). The marked heterogeneity in symptoms and severity across individuals renders diagnosis and clinical management particularly complex and challenging (4). Mounting evidence has highlighted the importance of early detection and subsequent intervention in optimizing outcomes. At present, evidence-based routine rehabilitations, such as Applied Behavior Analysis (ABA), speech therapy, and sensory integration training, have demonstrated substantial efficacy in mitigating symptoms (5). Nevertheless, these approaches may not fully address cognitive and psychological dimensions, prompting exploration of complementary strategies.

Creative play, encompassing imaginative, symbolic, and pretend play, is a natural, child-led way for children to explore emotions, roles, and social interactions. However, children with ASD frequently exhibit deficits in play skills due to communication difficulties, adaptive limitations, and restricted interests (6). Evidence indicates that play therapy is an effective and cost-efficient tool for managing children with ASD, improving their social communication and skills in a relaxed environment, and increasing parent–child interaction, with no significant side effects (7). A systematic review study indicates that play-based interventions yield medium-to-large effect sizes in improving core symptoms and daily functioning in children with ASD (8). Lee et al. (9) demonstrated that arranging play activities with missing items can enhance children’s symbolic play skills. The clinical trial by Kaur et al. (10) revealed that autistic children receiving creative yoga intervention exhibit improvement in social communication skills and joint attention. These findings highlight the promise of creative play in children with ASD. Nevertheless, most existing creative play interventions prioritize social or behavioral outcomes, with few incorporating cognitive functions like executive function or attention. Moreover, interventions often rely on structured play protocols, underemphasizing creative elements, which may undervalue the role of play intervention in fostering cognitive flexibility.

The study was conducted based on the theoretical framework of Naturalistic Developmental Behavioral Interventions (NDBIs), which integrate developmental and behavioral principles in naturalistic play contexts to address core ASD deficits (11). NDBIs emphasize child-initiated interactions, embedding learning opportunities in naturalistic environments, using behavioral strategies, and individualizing interventions based on the child’s developmental level and interests (12). These can enhance joint attention, imitation, turn-taking, and social motivation, which are pivotal skills that mediate broader improvements in social communication, maladaptive behaviors, and cognitive functioning in children with ASD. The principles of NDBIs align closely with the improved creative play intervention in this study, where activities are delivered in engaging play contexts (individualized and group-based), adapted weekly to the child’s interests and abilities, and incorporate adult scaffolding alongside child-led exploration. Conceptually, this intervention extends existing NDBI models (e.g., Joint Attention Symbolic Play Engagement and Regulation, JASPER; pivotal response training, PRT) by uniquely emphasizing creative multi-sensory activities and explicit group components to foster peer interaction.

The improved creative play intervention is clearly distinguished from standard play therapy. Standard play therapy is typically a non-directive, child-centered psychotherapeutic approach rooted in humanistic or psychoanalytic traditions. It emphasizes free emotional expression, trauma processing, and the therapeutic relationship through largely unstructured play, with minimal therapist direction, behavioral strategies, or explicit targeting of core ASD deficits such as joint attention and cognitive flexibility (13, 14). In contrast, the present intervention applies a structured yet naturalistic NDBI framework to systematically address multiple developmental domains through targeted activities, thereby providing a mechanistic pathway for improvements in social communication, maladaptive behaviors, and cognitive functioning in children with ASD.

This study aims to address gaps by comprehensively evaluating the effects of creative play interventions with improved protocols in children with ASD. It was hypothesized that the observation group may have superior pre-post changes in social communication, behavioral, and cognitive function.

## Methods

### Study design

This was a parallel-group, single-blind, randomized controlled trial (RCT) conducted at the pediatric department of Nanyang First People’s Hospital (Henan, China) from November 2023 to December 2025. Written informed consent was obtained from the participants’ patients. This study was approved by the Institutional Review Board of The First People’s Hospital of Nanyang City (approval number: 2023-yxlllz; date: 10 Nov. 2023) and conducted in accordance with the Declaration of Helsinki.

### Participants

Eligible subjects were screened based on the following inclusion criteria: (1) aged 3–8 years; (2) a confirmed diagnosis of ASD according to the Diagnostic and Statistical Manual of Mental Disorders, Fifth Edition (DSM-5) criteria (15); (3) intelligence quotient (IQ) ≥ 70 via Wechsler Preschool and Primary Scale of Intelligence (WPPSI-IV); (4) parental ability to attend sessions/follow-ups. According to DSM-5 severity levels, all participants required support (Level 1) or substantial support (Level 2); with no participants meeting criteria for Level 3 (requiring very substantial support), primarily due to the inclusion criterion of IQ ≥ 70. Exclusion criteria are as follows: (1) comorbid severe conditions (e.g., epilepsy, genetic metabolic disorders); (2) participation in other play/psychological trials within 3 months; (3) sensory impairments (hearing/vision) affecting assessments. Families were instructed not to initiate new therapies or significantly alter routine treatments during the 12-week study period. Compliance was monitored via weekly parent-reported logs reviewed by the research team. Withdrawal criteria included serious adverse events, adherence <80%, or parental withdrawal.

The sample size was calculated using G*Power 3.1 software, based on the effect size (Cohen’s d = 0.72) from previous reports that examined the Social Responsiveness Scale (SRS) in ASD children following play interventions (16). With a significance criterion (*α*) of 0.05 and a power of 80%, the required sample size was 32 per group. Considering a dropout rate of 10%, 36 participants per group were recruited for this study (72 in total).

### Randomization and blinding

Block randomization was conducted using computer-generated sequences in a 1:1 allocation ratio with variable block sizes. The randomization process was implemented by an independent investigator not involved in participant recruitment, intervention delivery, or outcome assessment. The allocation sequences were concealed using sequentially numbered, opaque, sealed envelopes. Envelopes were stored and managed by an independent research coordinator not involved in the study and were only opened when participants were enrolled. Due to the nature of interventions, intervention providers and parents were unblinded to group assignment to facilitate proper delivery and participation. However, all outcome assessors for clinician-observed measures, as well as data analysts, remained blinded to group allocation until database lock.

### Intervention delivery

Participants were assigned to either the control or intervention group. Both groups received routine rehabilitation training 5 days a week for 2 h/day over 12 weeks, including ABA, speech therapy, and sensory integration training (40–45 min/session).

The control group received routine rehabilitation training alone. For the intervention group, participants received improved creative play interventions in addition to the routine rehabilitation training. Improved creative play interventions included individualized (1:1 child-therapist/parent) and group-based (one therapist with 4–6 children per group) training activities, delivered on the same 5 days per week. Consequently, the intervention group received an additional 60–70 min of therapeutic contact time per day compared with the control group. Activities were selected and adapted weekly based on the child’s developmental level and interests.

Individualized sessions were conducted in a quiet, distraction-free room, consisting of the following six activities. (1) Hand-slap reaction game: The parent and child faced each other with palms hovering and counted down “3-2-1” together. On “1,” one attempted to lightly slap the back of the other’s hand, while the targeted person must quickly withdraw their hand to avoid being slapped. (2) Stacking cups or nesting toy sets: The parent/therapist first demonstrated how smaller containers fit inside larger ones. The child was then encouraged to imitate and independently nest or stack the cups/toys. (3) Building blocks: The parent and child jointly built structures using soft blocks. Upon completion, the child was encouraged to knock the structure down. (4) Mystery bag guessing: Safe, everyday objects with distinct textures, shapes, and functions were placed in an opaque drawstring bag. The child reached in without looking, felt the object, and attempted to guess what it was. Objects with potential hazards like keys, coins, pens, and hairpins were strictly excluded for safety. (5) Sorting practice: Various safe household items like socks, books, towels, cups, and snacks were presented. The child was allowed to sort them freely according to their own criteria. The parent/therapist then gently introduced alternative sorting strategies, such as by color, function, or ownership. (6) Ribbon threading: Ribbons and cardboard with pre-punched holes were prepared. The child first threaded the ribbon through the cardboard holes and then pulled the ribbon back out. These individualized activities lasted approximately 35–40 min per session.

Group-based training activities include: (1) High-five clapping game: The therapist played music with gradually increasing tempo (from slow to fast). Children clap to the beat independently or high-five parents or peers. During the activity, parents (guided by the therapist) dynamically change hand positions to enhance children’s visual tracking, observational skills, and sustained attention. (2) Music shake: The therapist played music while the child and parent held shakers, dancing, swaying, and shaking to the rhythm. (3) Drumming and percussion: Safe, unbreakable kitchen items (pots, pans, wooden spoons) were arranged as percussion instruments. A marching band video was shown as a model. Parents demonstrated striking techniques, encouraging children to freely bang and play. Empty paper towel rolls were used as “trumpets” to imitate brass instruments. (4) Free drawing: Non-toxic crayons, paints, stickers, and large sheets of paper were provided. Parents verbally labeled colors, shapes, and patterns while children freely drew. Upon completion, therapists and parents offered specific praise and encouragement. (5) Cooperative transport task: Children worked in pairs (child–child or child–parent) to transport a plastic bottle suspended by strings or ropes to a target basket without dropping it. Distance and difficulty were adjusted according to each child’s motor and social abilities. These activities were conducted in a large, safe activity room with soft flooring. Each group session lasted approximately 25–30 min, with transitions facilitated by visual schedules and songs to maintain engagement and reduce anxiety.

**Figure 1:**
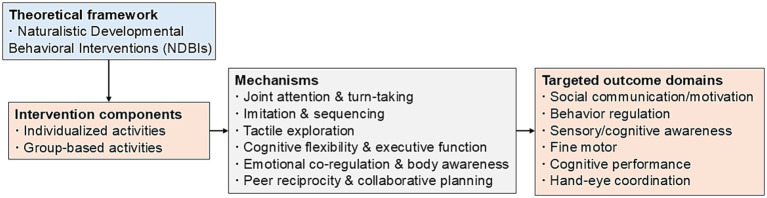
Conceptual model of the improved creative play intervention. The model illustrates how the individualized and group-based creative activities activate hypothesized mechanisms to improve targeted outcome domains.

The activities were designed to target specific deficits assessed by the outcome measures through mechanisms such as scaffolding joint attention, emotional co-regulation, and promotion of symbolic and flexible thinking (detailed in Table 1). A conceptual model of the intervention is presented in Figure 1.

**Table 1:** Alignment of creative play activities with targeted outcomes and hypothesized mechanisms.

Activities	Target domain (outcome measure)	Hypothesized mechanism
Hand-slap reaction game	Social communication (SRS-2), Behavioral regulation (ABC)	Promotes joint attention, turn-taking, and rapid motor response
Stacking cups/nesting toy sets, Building blocks	Cognitive performance, Imitation, Fine motor (C-PEP-3), Sensory/cognitive awareness (ATEC)	Encourages imitation, sequencing, problem-solving, and tolerance of change
Mystery bag guessing	Sensory/cognitive awareness (ATEC), Perception (C-PEP-3)	Stimulates tactile exploration, descriptive language, and imaginative thinking
Sorting practice	Cognitive performance (C-PEP-3), Social motivation (SRS-2)	Enhances cognitive flexibility, executive functioning, and verbal reasoning
Ribbon threading	Fine motor, Hand-eye coordination (C-PEP-3)	Improves motor planning and persistence
High-five clapping game	Social communication/motivation (SRS-2), Sociability (ATEC)	Enhances visual tracking, peer interaction, and sustained shared attention
Music shake	Sensory integration, Behavioral regulation (ABC)	Facilitates emotional co-regulation and body awareness via rhythm
Drumming and percussion	Social communication (SRS-2), Health/physical/behavior (ATEC)	Encourages spontaneous imitation, joint rhythm-making, and creative expression
Free drawing	Cognitive performance (C-PEP-3), Social motivation (SRS-2)	Promotes symbolic representation and verbal sharing
Cooperative transport task	Sociability (ATEC), Social impairment (CARS-2)	Requires joint planning, verbal/nonverbal coordination, and collaborative problem-solving

### Treatment fidelity

All therapists and participating caregivers completed a standardized two-day training workshop conducted by the principal investigator before the study began. A detailed intervention manual was provided, outlining activity procedures, weekly adaptation criteria based on child progress, and safety guidelines. Throughout the 12-week intervention, 20% of sessions (randomly selected, covering both individualized and group formats) were directly observed by an independent trained researcher using a structured fidelity checklist. The checklist assessed adherence to the protocol regarding activity sequence, individualization, safety measures, and positive reinforcement strategies. Mean fidelity was ≥90%. Inter-observer reliability was established on 10% of observed sessions with a second independent observer, yielding substantial agreement (Cohen’s kappa = 0.86).

### Outcome measures

Outcome measure assessments were conducted at T0 (baseline) and T1 (after 12-week intervention) by blinded evaluators in a quiet room. All clinician-administered assessments were performed by independent blinded evaluators (trained clinicians or research assistants who were not involved in intervention delivery and remained blind to group allocation throughout the study). Parents or primary caregivers, who were not blinded to group assignment due to their participation in the intervention, provide proxy reports for caregiver-rated measures. The following assessment tools were selected for their established reliability and validity in Chinese populations, wide use in ASD intervention research, comprehensive coverage of core domains, and demonstrated sensitivity to treatment-related changes. Specifically, the primary outcome measure is social communication assessed by the Social Responsiveness Scale, Second Edition (SRS-2), which has been reported to exhibit excellent psychometric properties (17). The SRS-2 has 65 items measured on a Likert scale from 0 (not true) to 3 (almost always true) and was completed by parents or primary caregivers. It consists of 5 subscales: social awareness, social cognition, social communication, social motivation, and autistic mannerisms. The Chinese version of the SRS-2 scale has good reliability and validity (17). Higher scores on the SRS-2 indicate more severe problems.

Secondary outcome measures are as follows:

(1) Behavior: Autism Behavior Checklist (ABC) is a caregiver-rated instrument completed by parents or primary caregivers, with 57 items on a 4-point Likert scale, assessing 5 domains: sensory, motor, social communication, language, and self-care ability. It can reflect the core symptoms of autism, and the Chinese version exhibits good reliability and validity (18). Higher ABC scores indicate more severe autistic behavioral symptoms.

(2) ASD severity: Childhood Autism Rating Scale, Second Edition (CARS-2) is a clinician-observed rating scale completed by blinded evaluators, with good reliability and validity (19). It evaluates ASD symptom severity across three domains, including social impairment, negative emotionality, and distorted sensory response, consisting of 15 items. Each item is scored 1–4 (1 = normal to 4 = severely abnormal), yielding a total score of 15–60. The total score <30 indicates no ASD, 30–36 indicates mid-to-moderate ASD, and >36 indicates severe ASD.

(3) Cognitive function: The Chinese version of Psychoeducational Profile-Third Edition (C-PEP-3) is a direct assessment tool administered and scored by blinded evaluators to evaluate developmental/cognitive functions in ASD (20). The C-PEP-3 comprises 12 subdomains, which are divided into two parts: the pathological behavior scale and the functional development scale. The functional developmental scale comprises seven subdomains, including imitation, perception, gross motor, fine motor, hand-eye coordination, verbal cognition, and cognitive performance, with scoring criteria as pass (P), emerge (E), and fail (F). Lastly, the child’s developmental level was determined based on the number of “P” in each developmental area corresponding to the developmental age.

(4) Overall symptoms: Autism Treatment Evaluation Checklist (ATEC) is a caregiver-rated questionnaire completed by parents or primary caregivers specifically used to evaluate changes in ASD severity in response to treatment. It consists of 77 items and is divided into four subscales: speech/language/communication (14 items), sociability (20 items), sensory/cognitive awareness (18 items), and health/physical/behavior (25 items) (21). The total score is calculated by summing the subscale scores, yielding a range of 0 to 179 points. Lower scores indicate milder ASD symptoms, whereas higher scores reflect greater severity.

### Data collection and management

Data were captured via electronic case report forms (eCRFs), with dual-entry verification. Demographic characteristics included age, sex, and parental education level. All participants were assigned identification code numbers. Upon recording eCRFs, personal identifying information (e.g., name, phone number, address, medical record number) was removed from the data. The data manager created and stored a linkable, anonymized number table.

### Statistical analysis

All analyses were performed adhering to intent-to-treat (ITT) principles. Data were analyzed using SPSS 27.0 (IBM, Armonk, NY, United States). The Shapiro–Wilk test was used to assess normality. Categorical variables are expressed as numbers and percentages (*n*[%]), and continuous variables are presented as mean ± standard deviation (SD). Between-group comparisons of baseline characteristics were performed using the Chi-square test or Fisher’s exact test for categorical variables and independent-samples t-tests for continuous variables. The *p*-value <0.05 indicated statistical significance.

The effects of the intervention on primary and secondary outcomes were evaluated using linear mixed-effects models (LMMs). These models accounted for repeated measures (baseline and 12-week follow-up) by including a random intercept for participants and fixed effects for group (intervention vs. control), time (pre- vs. post-intervention), and their interaction (group × time). The inclusion of a random intercept for each participant accounted for individual variability at baseline levels and response trajectories, which is particularly relevant given the heterogeneous presentation of symptoms and developmental profiles in children with ASD. Baseline outcome scores were used as covariates to adjust for potential imbalances. Model fit was assessed using Akaike Information Criterion (AIC) and restricted maximum likelihood. Estimated marginal means (*β*) were derived for group differences in change scores (T1 – T0), with 95% confidence intervals (CIs). No adjustment for multiple comparisons was performed for the secondary outcomes, as the study was primarily powered for the SRS-2, and secondary analyses were not intended to provide confirmatory evidence of treatment efficacy. Therefore, *p*-values for secondary outcomes are nominal (unadjusted) and should be interpreted with caution as hypothesis-generating rather than definitive.

## Results

### Demographic characteristics of the participants

**Figure 2:**
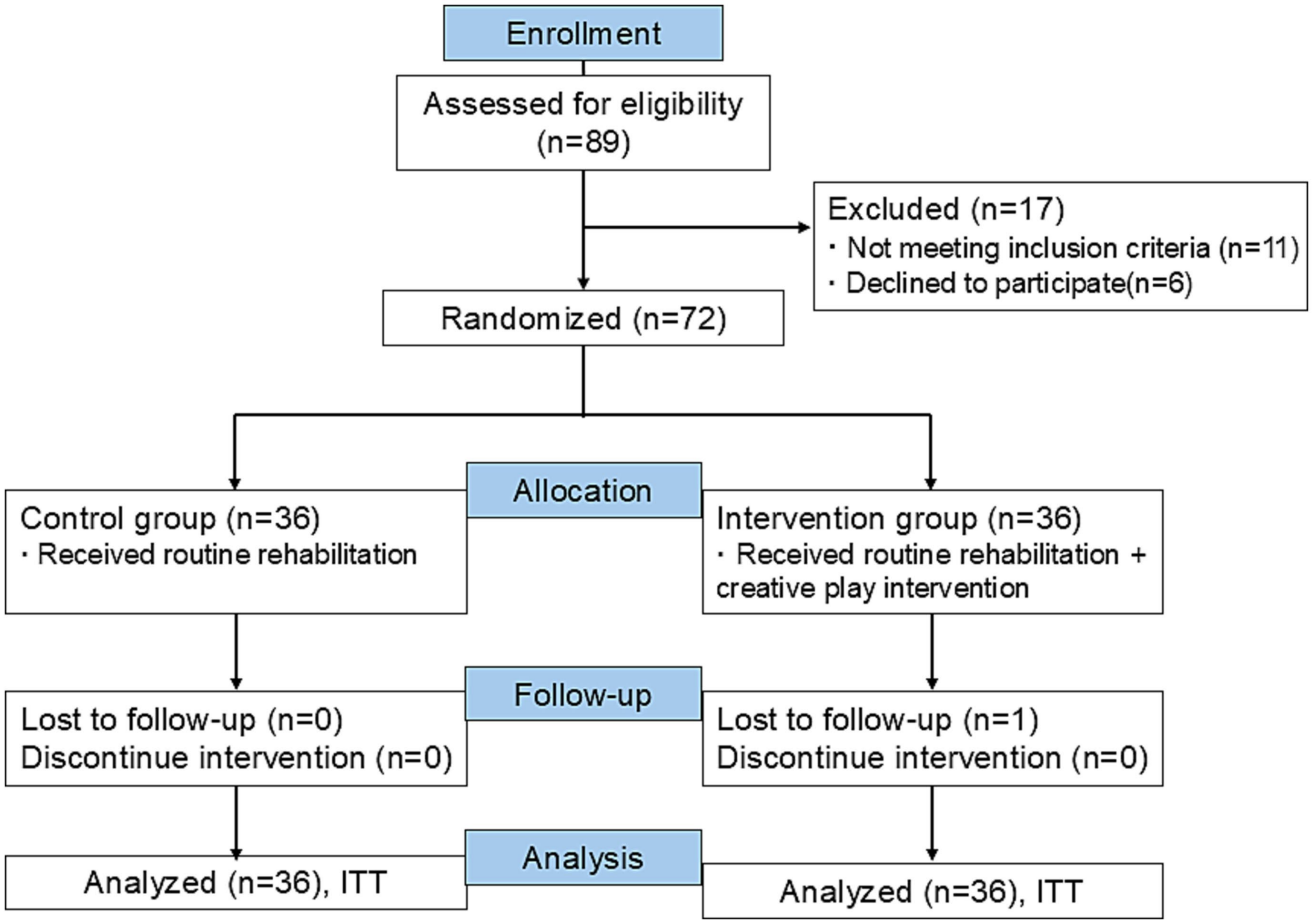
The flowchart of the study.

The flowchart of the study is shown in Figure 2. Of the 89 individuals initially screened, 72 were eligible and randomized into the control (*n* = 36) and intervention (*n* = 36) groups. The mean age of the patients was 4.56 ± 1.34 years, and the majority of them were males (79.17%). Baseline demographics showed no significant group differences (Table 2). Moreover, baseline ASD severity, as measured by CARS-2 total score, was also comparable between control and intervention groups (*p* = 0.18). These indicate successful randomization and minimization of potential confounding effects by symptom severity. Additionally, no severe adverse events were observed.

**Table 2:** Demographic characteristics of the two groups.

Variables	Control group (*n* = 36)	Intervention group (*n* = 36)	*p*-value
Age (years), mean ± SD	4.78 ± 1.39	4.35 ± 1.29	0.33
Sex, *n*(%)			0.56
Male	27(75.00%)	30(83.33%)	
Female	9(25.00%)	6(16.67%)	
Caregiver education level, *n*(%)			0.83
Below high school	13	12	
High school	10	9	
University or above	13	15	
CARS total score	53.60 ± 4.88	50.95 ± 7.18	0.18

### Primary outcome measure

**Figure 3:**
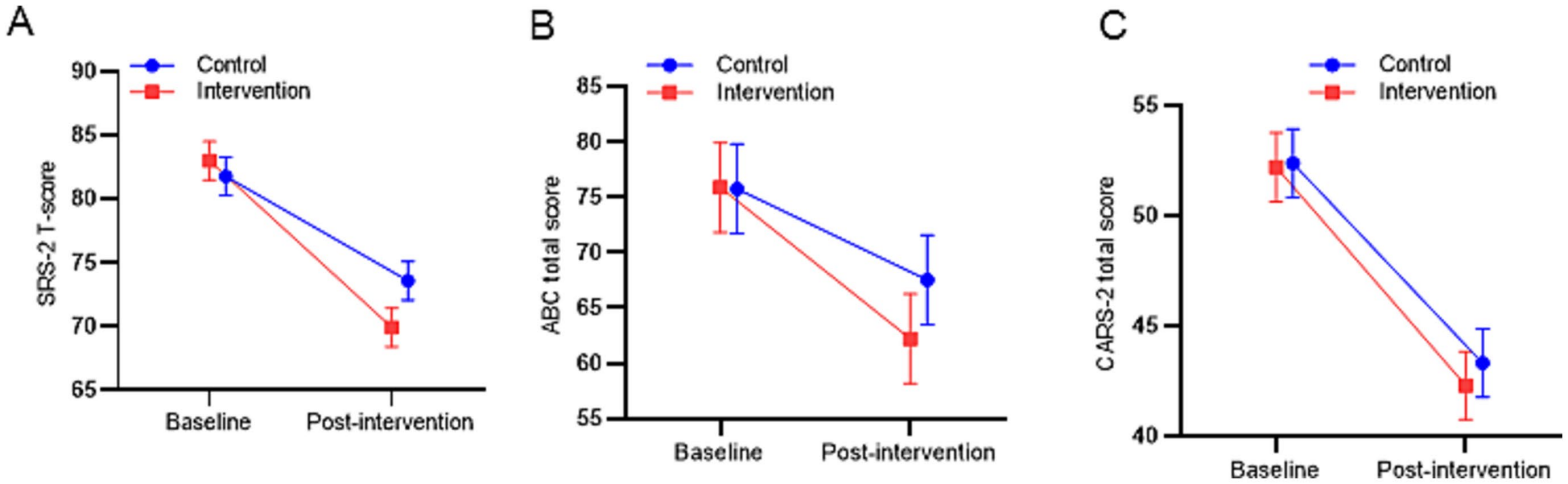
Line graphs show the estimated marginal mean values of SRS-2T-score **(A)**, ABC score **(B)**, and CARS-2 score **(C)** in the intervention and control groups at baseline and post-intervention after adjustment for baseline outcome scores. Error bars represent 95% confidence intervals (CI).

Table 3 presents the descriptive statistics for SRS-2T-scores at baseline, post-treatment, and changes, along with the results of the LMM analysis. Both groups exhibited significant declines in SRS-2T-scores (time main effect: *β* = 13.07 for baseline vs. post-intervention, *p* < 0.001), indicating an overall improvement in social communication symptoms following the 12-week intervention. In the control group, scores decreased from baseline 83.45 ± 10.67 to post-treatment 75.25 ± 12.45 (change −8.20 ± 3.86); in the intervention group, from 81.05 ± 9.75 to 68.53 ± 8.59 (change −12.52 ± 3.27). Figure 3A shows the estimated marginal mean of the two groups after adjustment for the baseline SRS-2T-score. The group × time interaction effect showed a greater improvement magnitude in the intervention group (*β* = −4.87, 95% CI [−7.48, −2.26], *p* = 0.001), suggesting potential advantages of the improved creative play intervention in enhancing social communication function.

**Table 3:** Changes in primary outcome SRS-2 T-scores in the control and intervention groups.

Time points	Control group (*n* = 36)	Intervention group (*n* = 36)	LMM *p*-value (time effect)	LMM *p*-value (group × time interaction)	*β* (95% CI)
Baseline (T0), mean ± SD	83.45 ± 10.67	81.05 ± 9.75	<0.001	0.001	−4.87(−7.48, −2.26)
Post-intervention (T1), mean ± SD	75.25 ± 12.45	68.53 ± 8.59			
Change (T1-T0)	−8.20 ± 3.86	−12.52 ± 3.27			

Data are descriptive statistics from observed values (mean ± SD). Negative changes indicate symptom improvement. LMM, linear mixed effects model (adjusted for baseline SRS-2 T-score). *β* indicates the adjusted between-group mean difference in change scores (intervention minus control).

### Secondary outcome measures

For the secondary outcome ABC and CARS-2 total scores (Table 4), LMM results revealed a significant main effect of time (ABC: *β* = 13.65 for baseline vs. post-intervention, *p* < 0.001; CARS-2: *β* = 9.9, *p* < 0.001), indicating overall reductions in ASD behavior and severity across both groups from baseline to post-intervention (Figures 3B,C). For ABC scores, the control group decreased from baseline 75.55 ± 5.02 to post-treatment 67.35 ± 11.52 (change −8.2 ± 10.9); the intervention group from 76.05 ± 13.60 to 62.40 ± 13.45 (change −13.65 ± 14.43). Notably, the interaction effect was not statistically significant (*β* = −5.45, 95% CI [−13.64, 2.74], *p* = 0.186), indicating similar behavioral symptom improvements across groups, although the intervention group exhibited a slightly larger change magnitude. For CARS−2 scores, within-group changes were −9.05 points for control (53.60 ± 4.88 vs. 44.55 ± 7.96) and −9.90 points for intervention (50.95 ± 7.18 vs. 41.05 ± 6.04). Critically, the group × time interaction was also non-significant (*β* = −0.85, *p* = 0.569), indicating no differential intervention effect on CARS score trajectories between groups.

**Table 4:** Changes in secondary outcome measures (ABC and CARS total scores) in the control and intervention groups.

Outcome	Control group (*n* = 36)	Intervention group (*n* = 36)	LMM *p*-value (time effect)	LMM *p*-value (group × time interaction)	*β* (95% CI)
ABC total score					
Baseline (T0), mean ± SD	75.55 ± 5.02	76.05 ± 13.60	<0.001	0.186	−5.45 (−13.64, 2.74)
Post-intervention (T1), mean ± SD	67.35 ± 11.52	62.40 ± 13.45			
Change (T1-T0)	−8.20 ± 10.90	−13.65 ± 14.43			
CARS-2 total score					
Baseline (T0), mean ± SD	53.60 ± 4.88	50.95 ± 7.18	<0.001	0.569	−0.85 (−3.85, 2.15)
Post-intervention (T1), mean ± SD	44.55 ± 7.96	41.05 ± 6.04			
Change (T1-T0)	−9.05 ± 5.31	−9.90 ± 3.96			

Data are descriptive statistics from observed values (mean ± SD). Negative changes indicate symptom improvement. LMM, linear mixed effects model (adjusted for baseline ABC or CARS-2 score). *β* indicates the adjusted between-group mean difference in change scores (intervention minus control).

**Figure 4:**
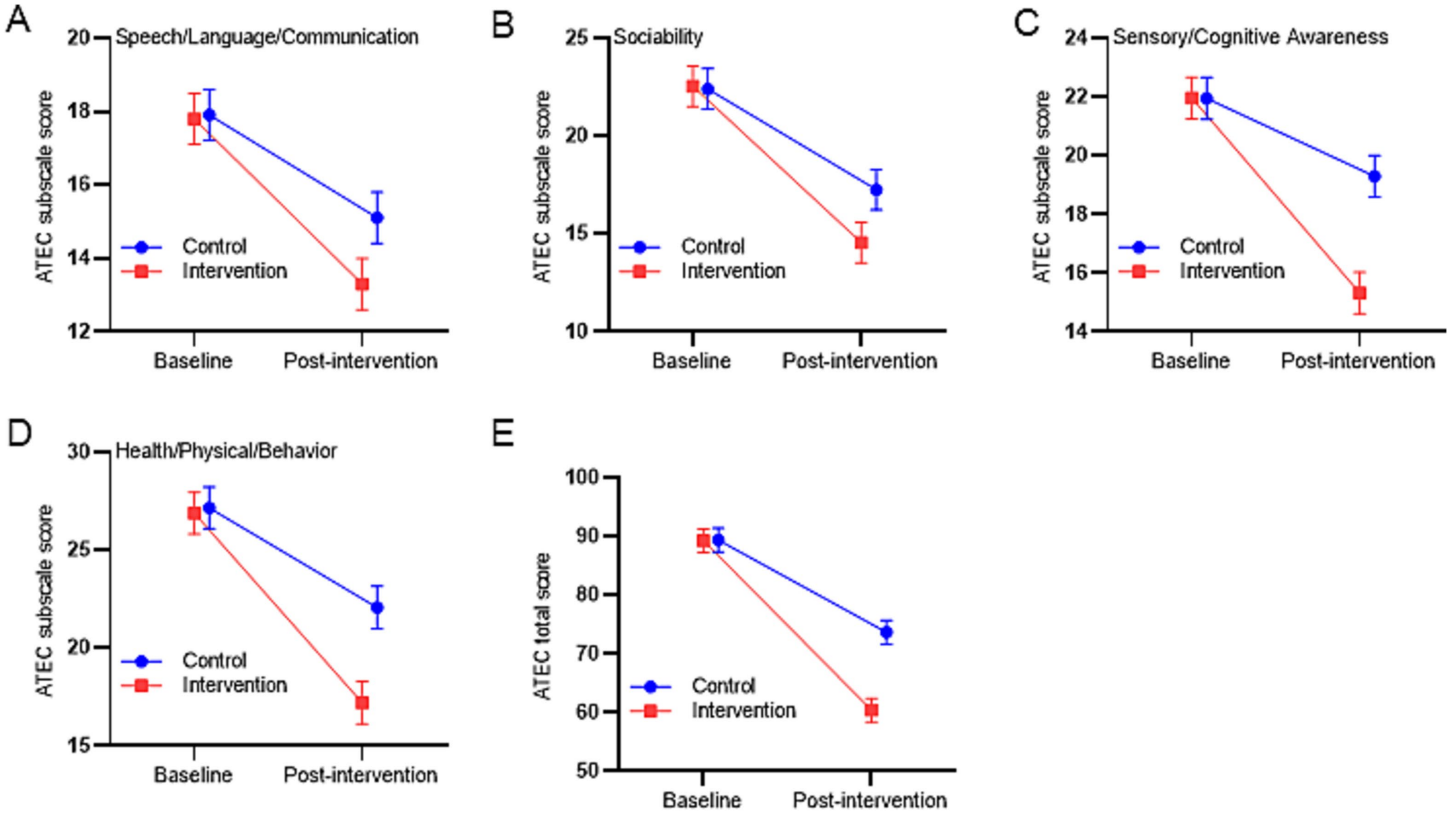
Line graphs show the estimated marginal mean values of ATEC subscales **(A–D)** and total score **(E)**. Error bars represent 95% confidence intervals (CI).

The ATEC evaluated changes in children with ASD across four domains. Table 5 presents the baseline (T0) and post-intervention (T1) descriptive statistics for each subscale and total score, along with group-specific changes (T1 − T0). LMM analysis revealed significant time effects across all subscales and the total score (*p* < 0.001), indicating overall symptom improvement following the 12-week intervention. Moreover, significant group × time interaction effects were observed for all subscales and the total score (*p* < 0.05), suggesting greater improvements in the improved creative play intervention group compared to the control group. The intervention group showed significantly larger reductions than the control group in speech/language/communication (*β* = −1.70, 95% CI [−3.14, −0.26], *p* = 0.022), sociability (*β* = −2.85, 95% CI [−5.02, −0.68], *p* = 0.012), sensory/cognitive awareness (*β* = −4.00, 95% CI [−5.42, −2.58], *p* < 0.001), and health/physical/behavior problems (*β* = −4.60, 95% CI [−6.91, −2.28], *p* < 0.001) (Figures 4A–D). Critically, a significant between-group difference was observed on the ATEC total score (*β* = −13.15, 95% CI [−17.36, −8.94], *p* < 0.001). After adjustment for the baseline score, the mean total ATEC score at week 12 was 60.40 (95% CI [58.39, 62.40]) in the intervention group versus 73.65 (95% CI [71.65, 75.66]) in the control group (Figure 4E), corresponding to a mean reduction of 28.85 points in the intervention group compared with 15.70 points in the control group.

**Table 5:** Changes in secondary outcome measures (ATEC and subscales) in the control and intervention groups.

Outcome	Control group (*n* = 36)	Intervention group (*n* = 36)	LMM *p*-value (time effect)	LMM *p*-value (group × time interaction)	*β* (95% CI)
Speech/language/communication					
Baseline (T0), mean ± SD	18.15 ± 3.22	17.55 ± 3.03	<0.001	0.022	−1.70 (−3.14, −0.26)
Post-intervention (T1), mean ± SD	15.35 ± 2.81	13.05 ± 2.72			
Change (T1-T0)	−2.80 ± 1.61	−4.50 ± 2.74			
Sociability					
Baseline (T0), mean ± SD	22.20 ± 3.02	22.75 ± 2.90	<0.001	0.012	−2.85 (−5.02, −0.68)
Post-intervention (T1), mean ± SD	17.05 ± 3.00	14.75 ± 3.65			
Change (T1-T0)	−5.15 ± 3.28	−8.00 ± 3.51			
Sensory/cognitive awareness					
Baseline (T0), mean ± SD	21.55 ± 5.75	22.35 ± 2.66	<0.001	<0.001	−4.00 (−5.42, −2.58)
Post-intervention (T1), mean ± SD	18.9 ± 6.43	15.70 ± 2.54			
Change (T1-T0)	−2.65 ± 1.57	−6.65 ± 2.72			
Health/physical/behavior					
Baseline (T0), mean ± SD	27.7 ± 3.75	26.35 ± 6.00	<0.001	<0.001	−4.60 (−6.91, −2.28)
Post-intervention (T1), mean ± SD	22.6 ± 3.34	16.65 ± 4.97			
Change (T1-T0)	−5.1 ± 2.94	−9.70 ± 4.19			
ATEC total score					
Baseline (T0), mean ± SD	89.60 ± 7.77	89.00 ± 6.64	<0.001	<0.001	−13.15 (−17.36, −8.94)
Post-intervention (T1), mean ± SD	73.9 ± 7.38	60.15 ± 7.92			
Change (T1-T0)	−15.7 ± 6.58	−28.85 ± 6.58			

Data are descriptive statistics from observed values (mean ± SD). Negative changes indicate symptom improvement. LMM, linear mixed effects model (adjusted for baseline scores of each subscale). *β* indicates the adjusted between-group mean difference in change scores (intervention minus control). Adjustment for multiple comparisons was performed.

The C-PEP-3 functional development scales assess seven domains using a pass (P)/emerge/fail scoring system. Higher numbers of passed items reflect higher developmental levels. No significant baseline differences were observed between groups on any C-PEP-3 subscale (all *p* > 0.05). Within-group difference analysis revealed significant improvements from baseline to week 12 in nearly all domains in both groups, except Gross motor in the control group (*p* = 0.085). Between-group comparisons of post-intervention scores and change scores showed that the improved creative play intervention group achieved significantly greater gains in the majority of developmental domains (Table 6). Specifically, the intervention group demonstrated significantly larger improvements than the control group in Fine motor (mean change 2.85 ± 2.28 vs. 1.30 ± 1.56, *p* = 0.002), Hand-eye Coordination (3.00 ± 2.17 vs. 1.80 ± 1.57, *p* = 0.009), Cognitive performance (3.10 ± 1.95 vs. 1.05 ± 2.28, *p* < 0.001), and Verbal cognition (1.49 ± 1.08 vs. 0.90 ± 0.35, *p* = 0.003). Trends toward superior gains in the intervention group were also observed in Imitation (*p* = 0.056) and Perception (*p* = 0.067).

**Table 6:** Changes in secondary outcome measures (C-PEP-3 subscales) in the control and intervention groups.

Outcome	Control group (*n* = 36)	Intervention group (*n* = 36)	Between-group *p*-value
Imitation (P)			
Baseline (T0), mean ± SD	2.95 ± 2.91	2.55 ± 1.23	0.256
Post-intervention (T1), mean ± SD	4.06 ± 1.21	4.50 ± 0.63	
Change (T1-T0)	1.66 ± 1.12	1.95 ± 1.03	
Within-group *p*-value	0.038	<0.001	
Perception (P)			
Baseline (T0), mean ± SD	4.80 ± 2.95	5 ± 3.51	0.617
Post-intervention (T1), mean ± SD	6.65 ± 2.81	7.05 ± 3.08	
Change (T1-T0)	1.85 ± 1.53	2.05 ± 1.84	
Within-group *p*-value	0.008	0.010	
Fine motor (P)			
Baseline (T0), mean ± SD	3.30 ± 2.61	3.45 ± 2.76	0.001
Post-intervention (T1), mean ± SD	4.60 ± 2.16	6.30 ± 2.40	
Change (T1-T0)	1.30 ± 1.56	2.85 ± 2.28	
Within-group *p*-value	0.024	<0.001	
Gross motor (P)			
Baseline (T0), mean ± SD	5.75 ± 3.46	5.75 ± 3.50	0.229
Post-intervention (T1), mean ± SD	7.1 ± 3.09	7.4 ± 3.10	
Change (T1-T0)	1.35 ± 1.08	1.65 ± 1.02	
Within-group *p*-value	0.085	0.038	
Hand-eye coordination (P)			
Baseline (T0), mean ± SD	3.40 ± 2.99	3.95 ± 3.34	0.009
Post-intervention (T1), mean ± SD	5.20 ± 2.96	6.95 ± 3.56	
Change (T1-T0)	1.80 ± 1.57	3.00 ± 2.17	
Within-group *p*-value	0.012	<0.001	
Cognitive performance (P)			
Baseline (T0), mean ± SD	1.8 ± 0.85	2.00 ± 1.03	<0.001
Post-intervention (T1), mean ± SD	2.85 ± 2.11	5.10 ± 2.21	
Change (T1-T0)	1.05 ± 2.28	3.10 ± 1.95	
Within-group *p*-value	0.007	<0.001	
Verbal cognition (P)			
Baseline (T0), mean ± SD	1.2 ± 0.72	1.25 ± 1.01	0.003
Post-intervention (T1), mean ± SD	2.1 ± 1.02	2.74 ± 2.03	
Change (T1-T0)	0.9 ± 0.35	1.49 ± 1.08	
Within-group *p*-value	<0.001	<0.001	

Data are descriptive statistics from observed values (mean ± SD). Positive changes indicate symptom improvement. Between-group differences using independent-samples *t*-tests. No adjustment for multiple comparisons was performed.

## Discussion

The present RCT demonstrated that a 12-week improved creative play intervention, alongside standard rehabilitation, led to significantly greater improvements in core and associated symptoms of ASD in young children compared to standard rehabilitation alone. The most pronounced effects were observed on overall symptom severity, as measured by the ATEC total score (group × time interaction *β* = −13.15, *p* < 0.001), with the intervention group achieving nearly double the reduction compared to the control group (mean change −28.85 vs. −15.70 points). The strongest subdomain effect emerged in sensory/cognitive awareness (*β* = −4.00, *p* < 0.001), alongside nominally significant benefits across sociability, speech/language, and health/physical/behavior subscales. Substantial gains were also evident in C-PEP-3 cognitive performance, fine motor, hand-eye coordination, and verbal cognition domains. These findings suggest that the improved creative play intervention particularly enhances sensory integration, cognitive flexibility, and adaptive behaviors, likely due to its multisensory, playful, and child-interest-driven activities.

A deficit in communication and reciprocal social interactions is one of the main characteristics of ASD (22). A systematic review of 32 RCTs underscored the versatility of play interventions in enhancing daily functioning, with 24 RCTs specifically targeting social and communicative outcomes (8). A scoping review also shows that play-based interventions improve social communication in autistic children aged 2–8 years (23). Consistent with these reports, our intervention produced significant advantages in social responsiveness (SRS-2) and multimodal ATEC domains, likely through activities promoting joint attention, turn-taking, and shared intentionality (e.g., cooperative transport tasks, high-five games, mystery bag guessing). The large treatment effect on the ATEC total score aligns with moderate-to-large effects reported in previous play-based and behavior trials (24), supporting the role of improved creative play interventions in naturally reinforcing sensory processing and reciprocal interaction.

In contrast, no significant group × time interactions were found on aberrant behavior (ABC) or overall autism severity (CARS-2). This dissociation between caregiver-reported and blinded clinician outcomes is a commonly observed pattern in short-duration behavioral trials (25, 26). It may also reflect several factors, including parents’ greater exposure to naturalistic changes, the 12-week treatment window’s insufficiency for detectable shifts in clinician-observed severity, or the CARS-2’s lower sensitivity to incremental improvements in higher-functioning children (27). Importantly, since significant improvements were primarily observed in caregiver-reported measures (SRS-2, ATEC), we cannot rule out the possibility of expectancy bias. Parents in the intervention group, aware of receiving the additional improved creative play intervention, may have rated their child’s behaviors more favorably due to heightened expectations. Additionally, the absence of significant effects on ABC and CARS-2 indicates that the benefits of the improved creative play interventions are primarily in specific caregiver-reported domains rather than broad multidimensional improvement.

A key advancement of this study lies in our design of “improved” creative play protocol, which combines structured elements with flexibility. We integrated individualized and small-group activities, adjusted weekly based on the child’s developmental level and interests. Unlike most previous play interventions focusing on social outcomes, our approach demonstrated broad cognitive and developmental benefits (C-PEP-3), suggesting the improved creative play as a complementary modality that targets executive function, symbolic representation, flexible problem-solving, and cooperative peer interaction. These are all recognized precursors of cognitive and language development in ASD (28, 29). Activities such as cooperative transport task, magic bag guessing, and collective music/drum play explicitly promoted turn-taking, shared intentionality, and perspective-taking, whereas individual activities (e.g., building blocks, ribbon threading, free drawing) promoted symbolic play and executive function. These domains are often delayed in ASD but highly responsive to play interventions (30, 31). Our results extend current understanding by showing that joy-based, multisensory play can augment standard early intervention regimens, potentially offering a more engaging and generalizable therapeutic mechanism.

The present study has both theoretical and practical implications for further research. The improved creative play intervention, applying the NDBI theoretical framework, confers high feasibility for real-world implementation. Activities utilize inexpensive, everyday materials (e.g., household items, ribbons, blocks) and require no specialized equipment. The short session duration (25–40 min), parent co-facilitation, and compatibility with existing rehabilitation schedules facilitate integration into community clinics, early intervention centers, or inclusive preschools. Additionally, brief therapist training, provided through workshops and manuals, further supports scalability.

It is important to acknowledge that our study still has some limitations. First, the moderate sample size (*n* = 72) may have limited power to detect smaller effects, particularly on clinician-rated measures. Second, the 12-week duration provides only short-term evidence; the absence of long-term follow-up assessments precludes conclusions about effect sustainability or maintenance. Third, the participants were predominantly young (3–8 years), male (79.17%), and verbally able with IQ estimates above 70, limiting generalizability to females, older children, minimally verbal individuals, or more intellectually impaired children. Fourth, due to additional therapeutic contact time in the intervention group, we cannot fully exclude the possibility that some observed improvements may be attributable to greater therapeutic exposure rather than to the specific effects of the improved creative play interventions. Nevertheless, this additive design reflects real-world clinical practice; future trials should employ dose-matched active controls to isolate the intervention’s unique contributions. Moreover, potential variability in diagnostic severity levels, lack of parent/therapist blinding (risking expectancy bias, especially for parent-rated outcomes), and inability to fully exclude minor unreported external influences (despite instructions and randomization) further temper interpretations. However, the randomized controlled design and parallel routine training substantially mitigate maturation and confounding risks. Future investigations should address these gaps through larger, multicenter RCTs with stratified randomization by baseline play skills, severity, and verbal ability. Incorporating 6–12-month follow-ups would clarify effect durability, while testing improved protocols in minimally verbal or intellectually impaired children could broaden applicability. Examining the optimal intensity, frequency, and duration of the intervention, along with treatment fidelity in community settings and neural mechanisms (e.g., via neuroimaging), would further refine this model.

In conclusion, our study suggests that improved creative play interventions, combined with routine rehabilitation training, may improve caregiver-reported social communication and aspects of cognitive-adaptive functioning in young children with ASD. Our findings provide preliminary support for incorporating creative play elements into standard early intervention regimens and highlight the potential of developmentally oriented, joy-based approaches to complement traditional behavioral therapies, although results should be interpreted cautiously in light of possible expectancy bias in parent reports.

## Data Availability

The original contributions presented in the study are included in the article/supplementary material, further inquiries can be directed to the corresponding authors.
