# Correction: Effects of improved creative play interventions on social communication, behavioral, and cognitive function in children with autism spectrum disorder: a randomized controlled trial

**DOI:** 10.3389/fpubh.2026.1856928

**Published:** 2026-04-27

**Authors:** Frontiers Production Office

**Affiliations:** Frontiers Media SA, Lausanne, Switzerland

**Keywords:** ASD, cognitive functioning, improved creative play intervention, randomized controlled trial, social communication

Author Shaohua Zhang was erroneously assigned as equal contributing author.

Author Yanfang Zhang was erroneously omitted as equal contributing author. The original version of this article has been updated.

